# Public Interest in Cosmetic Surgical and Minimally Invasive Plastic Procedures During the COVID-19 Pandemic: Infodemiology Study of Twitter Data

**DOI:** 10.2196/23970

**Published:** 2021-03-16

**Authors:** Wenhui Liu, Zhiru Wei, Xu Cheng, Ran Pang, Han Zhang, Guangshuai Li

**Affiliations:** 1 Plastic and Reconstructive Surgery First Affiliated Hospital Zhengzhou University Zhengzhou China

**Keywords:** COVID-19, Twitter, Google Trends, plastic procedure, trend, survey, surgery, social media

## Abstract

**Background:**

The unprecedented COVID-19 pandemic has brought drastic changes to the field of plastic surgery. It is critical for stakeholders in this field to identify the changes in public interest in plastic procedures to be adequately prepared to meet the challenges of the pandemic.

**Objective:**

The aim of this study is to examine tweets related to the public interest in plastic procedures during the COVID-19 pandemic and to help stakeholders in the field of plastic surgery adjust their practices and sustain their operations during the current difficult situation of the pandemic.

**Methods:**

Using a web crawler, 73,963 publicly accessible tweets about the most common cosmetic surgical and minimally invasive plastic procedures were collected. The tweets were grouped into three phases, and the tweeting frequencies and Google Trends indices were examined. Tweeting frequency, sentiment, and word frequency analyses were performed with Python modules.

**Results:**

Tweeting frequency increased by 24.0% in phase 2 and decreased by 9.1% in phase 3. Tweets about breast augmentation, liposuction, and abdominoplasty (“tummy tuck”) procedures consecutively increased over the three phases of the pandemic. Interest in Botox and chemical peel procedures revived first when the lockdown was lifted. The COVID-19 pandemic was associated with a negative impact on public sentiment about plastic procedures. The word frequency pattern significantly changed after phase 1 and then remained relatively stable.

**Conclusions:**

According to Twitter data, the public maintained their interest in plastic procedures during the COVID-19 pandemic. Stakeholders should consider refocusing on breast augmentation, liposuction, and abdominoplasty procedures during the current phase of the pandemic. In the case of a second wave of COVID-19, stakeholders should prepare for a temporary surge of Botox and chemical peel procedures.

## Introduction

The unprecedented COVID-19 pandemic has brought drastic changes not only to daily life but also to the field of plastic surgery [1-4]. Due to repeated periods of quarantine and the deteriorating economy, many people are not scheduling nonessential medical services, such as plastic procedures; this has resulted in concerning prospects for stakeholders in this field, including plastic surgeons, hospital administrators, and clinic owners. These stakeholders require a better understanding of the public interest in common plastic procedures to adjust their practices so that they can sustain operations during this difficult time.

This critical understanding could be obtained by a survey including as many people as possible; however, such a study would be both time-consuming and expensive. Therefore, we turned to infodemiology [5], which has been employed for a long time to monitor public health issues and people’s status [6,7]. During the current COVID-19 pandemic, numerous infodemiology studies have deepened our understanding of human behavior related to COVID-19 [8]. The social media platform Twitter is widely applied in the field of infodemiology [9,10]. In the field of plastic surgery, Twitter has been deployed to investigate public perception toward plastic surgery [11,12] and the engagement of plastic surgeons with social media [13,14]. Therefore, Twitter data can be used to examine public thoughts in a rapid and economic way. Thus, we collected tweets related to plastic procedures, and these data may reveal public interest in these procedures to some extent.

In this study, we extracted 73,963 publicly accessible tweets about the most common plastic procedures from January 1 to July 22, 2020. Tweeting frequency, sentiment, and word frequency analyses were applied to examine the changes in public interest in plastic procedures. This study may help stakeholders in the field of plastic surgery refocus their practices and sustain operations during the pandemic.

## Methods

### Data Acquisition

Tweets posted from January 1 to July 22, 2020 UTC were retrieved from Twitter using Locoyposter [15]. Locoyposter is a commercial web scraping tool that provides a visual interface that is friendly to users with little programming experience. It provides a framework in which users can collect data, such as posts on Twitter, by designing a workflow, parsing the target webpages, specifying XPath expressions, and storing the content in an external database. Keywords were determined by referring to the top plastic surgery procedures in the latest annual Plastic Surgery Statistics published by the American Society of Plastic Surgeons [16]. The top five cosmetic surgical procedures and the top five minimally invasive procedures were extracted. Among these procedures, the academic term *botulinum toxin type A* did not appear to be well known among the public, as it only returned a few tweets; therefore, the search term *Botox* was adopted because the commercial preparation Botox comprised the largest portion of botulinum toxin type A treatments according to the statistical report. The term *soft tissue fillers* was substituted with *hyaluronic acid* for the same reason.

This study focuses on firsthand and self-revealed interest in plastic procedures. Therefore, we further filtered the collected tweets by excluding replies and tweets with links. For tweets including links, interpretation should be made by referring to former tweets based on the specific conversation context or external webpages. These tweets are heterogeneous compared to their counterparts, which are self-explanatory. Introduction of these tweets would create uncertainty in this study. Only tweets in English were retained in the downstream analysis. The final query is shown below:

(“Breast Augmentation” OR Liposuction OR Rhinoplasty OR “Eyelid Surgery” OR “Tummy Tuck” OR “Botox” OR “Hyaluronic acid” OR “Chemical Peel” OR “Laser Hair Removal” OR Microdermabrasion) lang:en until:2020-07-22 since:2020-01-01 -filter:links -filter:replies.

This tweet search can be reproduced by pasting this query into the search box on Twitter.

We also searched these keywords on Google Trends [17], which has previously been applied in research on COVID-19 [18,19] and cosmetic procedures [20]. We searched these keywords as topics to include as many related searches as possible. The region was set as *worldwide* and the category as *all*. The default web search was selected, and the time span was January 1 to July 22, 2020. Because Google Trends normalizes data when multiple keywords are searched together [21], we searched each keyword separately. Google Trends identified “eyelid surgery” under the topic of blepharoplasty and “tummy tuck” under the topic of abdominoplasty. Therefore, these keywords were used for searches instead of the original keywords. Google Trends failed to identify any related topics for Botox; therefore, we used *Botox* as the only search term related to botulinum toxin type A. Visualization of the Google Trends of these keywords could aid division of the tweets into groups and understanding of the corresponding Twitter data.

### Tweeting Frequency

Tweeting frequency was used to indicate public interest in plastic procedures. It is clear that users may tweet more frequently about certain plastic procedures if they are interested in them. We compared the overall tweeting frequencies in different phases of the pandemic. The frequencies of specific procedures and constituent ratios for all procedures were also determined. Proportions of tweets that mentioned COVID-19 were also compared by calculating the co-occurrence rate of *covid* or *coronavirus* in the tweets.

### Sentiment Analysis

Sentiment analysis was performed using the Natural Language Toolkit (NLTK) [22]. We first tokenized the tweets and removed stop words and punctuation. Then, the sentiments were determined by the VADER (Valence Aware Dictionary and Sentiment Reasoner) module, which is specifically attuned to sentiments expressed in social media [23]. We compared sentiments among different phases. We also compared sentiment differences for tweets that mentioned COVID-19 to reveal overall sentiment changes caused by COVID-19. Tweets that mentioned COVID-19 were tagged if they contained *covid* or *coronavirus* in their text. Sentiment changes for specific procedures and overall sentiment constituent ratio changes were also determined.

### Word Frequency

Word frequency can reflect trends in topics on Twitter [24,25]. After tokenization and removal of stop words and punctuation, all words were transformed to lowercase, labeled with a part-of-speech tag, and lemmatized. Then, the word frequencies were counted and compared among different phases after normalization by the total number of tweets. A change in the frequently used words may reflect a change in public interest. Therefore, words with the highest change ratios were highlighted. These steps were achieved with the Python [26] modules NLTK and WordCloud. To better understand the word frequency results, we also provided some typical tweets for readers.

### Grouping and Statistical Analysis

**Figure 1:**
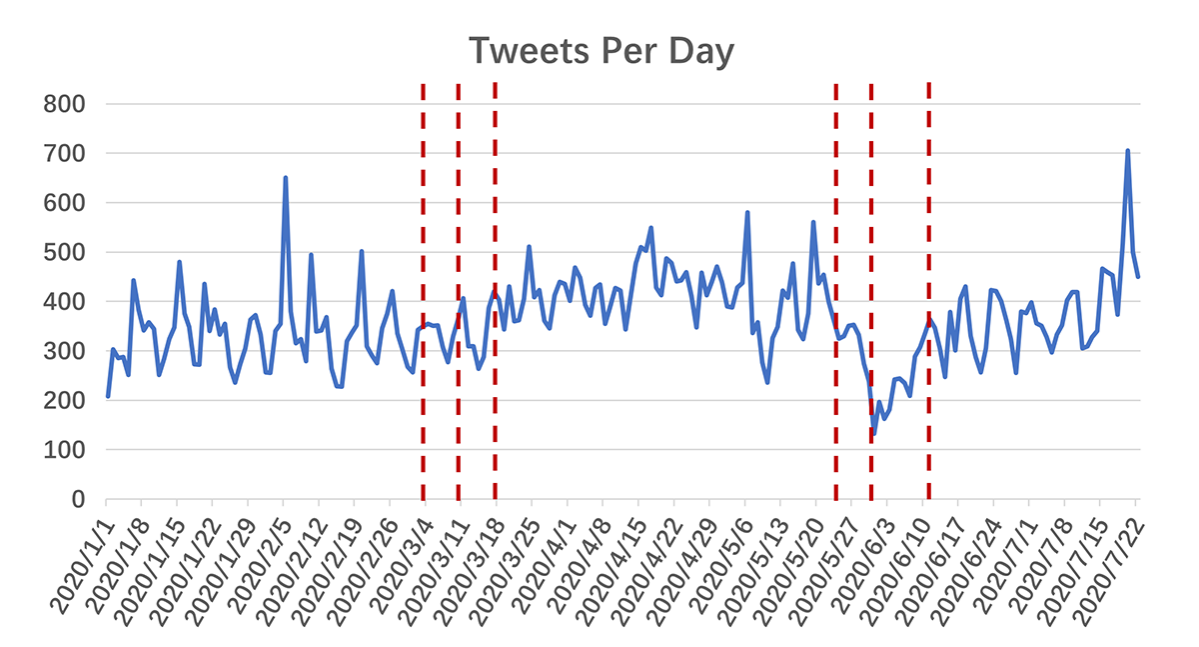
Frequency of tweets related to the top plastic procedures. The dashed red lines indicate landmark events and the days around them that were excluded from the data collection process.

The collected tweets were divided into three phases. After scrutinizing the data, we found that the tweeting frequency and Google Trends indices fluctuated drastically around two significant events. The first event is the declaration of COVID-19 as a pandemic by the World Health Organization (WHO) on March 11, 2020. After this event, the amount of public attention being paid to COVID-19 increased substantially. The second significant event is the death of George Floyd [27]. The public outcry against this event drew attention away from the pandemic for a period of time. Therefore, we excluded data around these two events and divided the tweets into three phases: phase 1 (January 1 to March 4, 2020), phase 2 (March 18 to May 24, 2020), and phase 3 (June 11 to July 22, 2020). More details are provided in Figure 1.

Quantitative variables were analyzed by one-way analysis of variance and qualitative variables were analyzed by chi-square tests using SPSS version 25 (IBM Corporation). Differences were considered significant at a *P* value of <.05.

## Results

### Tweeting Frequency

We retrieved 73,963 publicly accessible tweets about the most common plastic procedures from January 1 to July 22, 2020. We provide these tweets and their corresponding publication dates in Multimedia Appendix 1. Other information was not provided to protect the privacy of the tweet writers.

The tweeting frequency per day is shown in Figure 1. The figure shows that the tweeting frequency is different in each of the three phases. After the WHO declared COVID-19 a pandemic, the tweeting frequency increased (ie, the tweeting frequency in phase 2 is higher than that in phase 1). The tweeting frequency then decreased sharply after May 25, and the tweeting frequency in phase 3 is lower than that in phase 2. Some peaks can also be seen in the curve; these peaks were basically related to celebrities and to speculation that they may have undergone cosmetic procedures. For example, the peak on February 5 was related to Nancy Pelosi, and that on July 20 was related to Kamala Harris. More details are provided in Multimedia Appendix 1.

**Figure 2:**
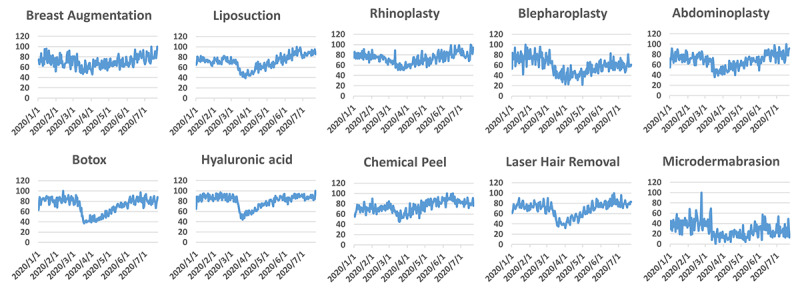
Google Trends indices for the 10 most common cosmetic surgical and minimally invasive plastic procedures. The searched topics are shown rather than the actual keywords.

We also searched the top plastic procedures with Google Trends (Figure 2). It can be inferred that searches related to these plastic procedures decreased sharply after the WHO declared COVID-19 a pandemic and rebounded slowly after that. Public searches related to the majority of these procedures did decrease at the end of May and the beginning of June. However, the decrease was less substantial than that of its Twitter counterpart.

**Figure 3:**
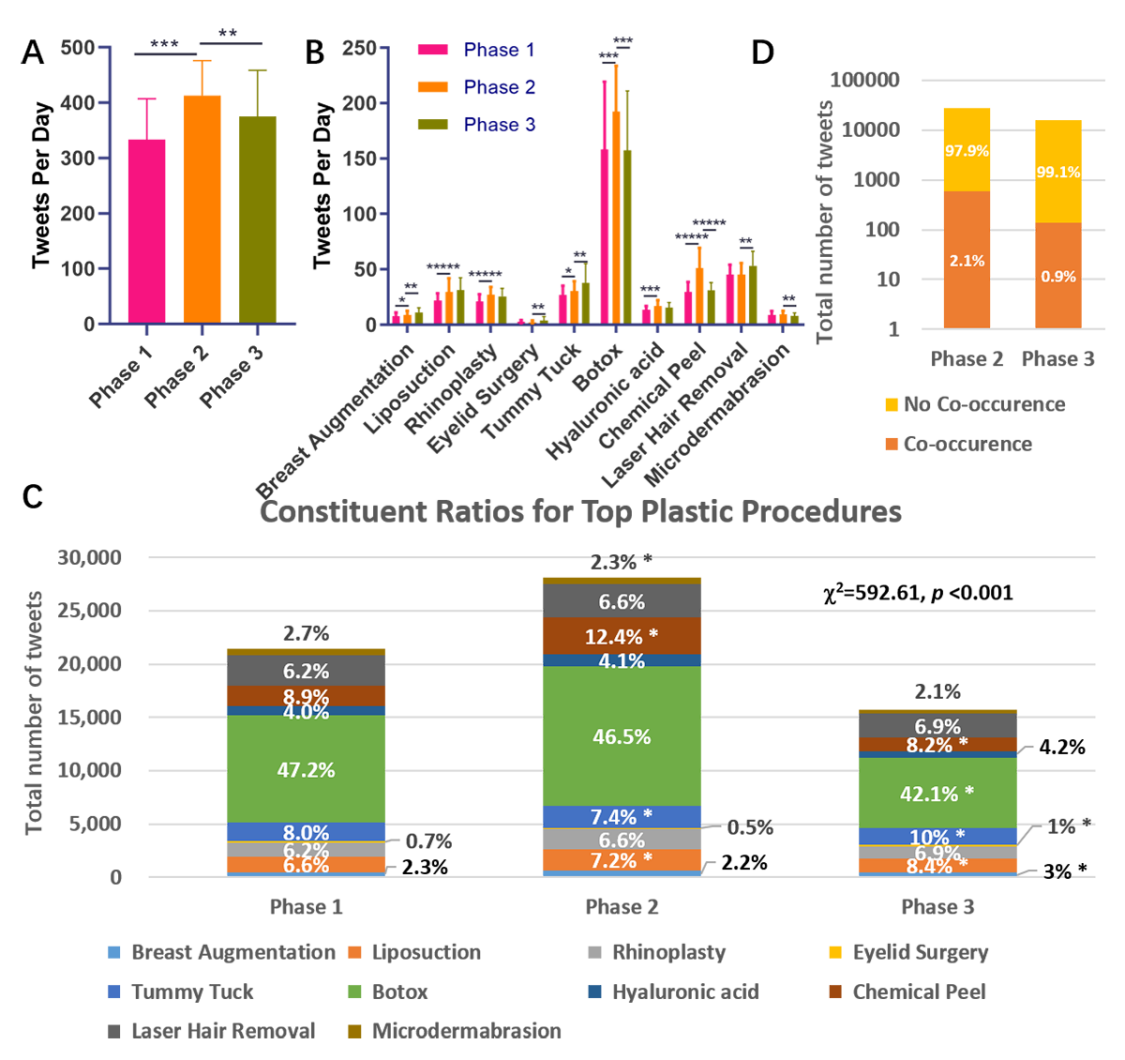
(A) Overall tweeting frequencies in each phase. (B) Tweeting frequencies for specific procedures. (C) Total numbers and constituent ratios (labels in cells) of tweeting frequency for the top plastic procedures. (D) Frequencies and constituent ratios (labels in cells) for tweets in which mentions of the procedures do and do not co-occur with mentions of COVID-19 in phases 2 and 3. *P<.05, **P<.01, ***P<.001.

Further statistical analysis was consistent with the results in Figure 1. As shown in Figure 3A, overall tweeting frequency per day in phase 2 increased by as much as 24.0% compared to phase 1 (333.17 vs 413.20), while tweeting frequency decreased by 9.1% after that (413.20 vs 375.33). When comparing the frequencies of tweets related to specific procedures in phase 2 and phase 1, most of them increased; only the tweeting frequencies for *eyelid surgery*, *laser hair removal*, and *microdermabrasion* showed no statistical difference. For phase 3 and phase 2, the frequency of tweets related to most procedures increased or remained stable, while the tweeting frequencies for *Botox*, *chemical peel*, and *microdermabrasion* decreased (Figure 3B).

In addition to the direct comparison of the tweeting frequencies, their relative changes were determined with a constituent ratio. As shown in Figure 3C, the overall constituent ratios were different in the different phases (χ^2^=592.61, *P*<.001). We further applied partition of the chi-square test to each specific procedure with *P* values adjusted by Bonferroni corrections using SPSS. It can be inferred that the constituent ratios of *liposuction* and *tummy tuck* increased in both phase 2 and phase 3. The constituent ratios of *breast augmentation*, *eyelid surgery*, and *laser hair removal* increased in phase 3 versus phase 2, while the constituent ratios of *Botox* and *chemical peel* decreased in phase 3 versus phase 2. It should be noted that one tweet may mention more than one plastic procedure; therefore, the sum of the frequencies in Figure 3C is slightly higher than the overall number of tweets in each phase.

The proportion of tweets mentioning COVID-19 can reflect public concern to some extent. As shown in Figure 3D, the percentage of tweets that mentioned COVID-19 decreased from 2.1 in phase 2 to 0.9 in phase 3. In phase 1, only a few tweets mentioned COVID-19. Therefore, the corresponding data were not analyzed.

### Sentiment Analysis

**Figure 4:**
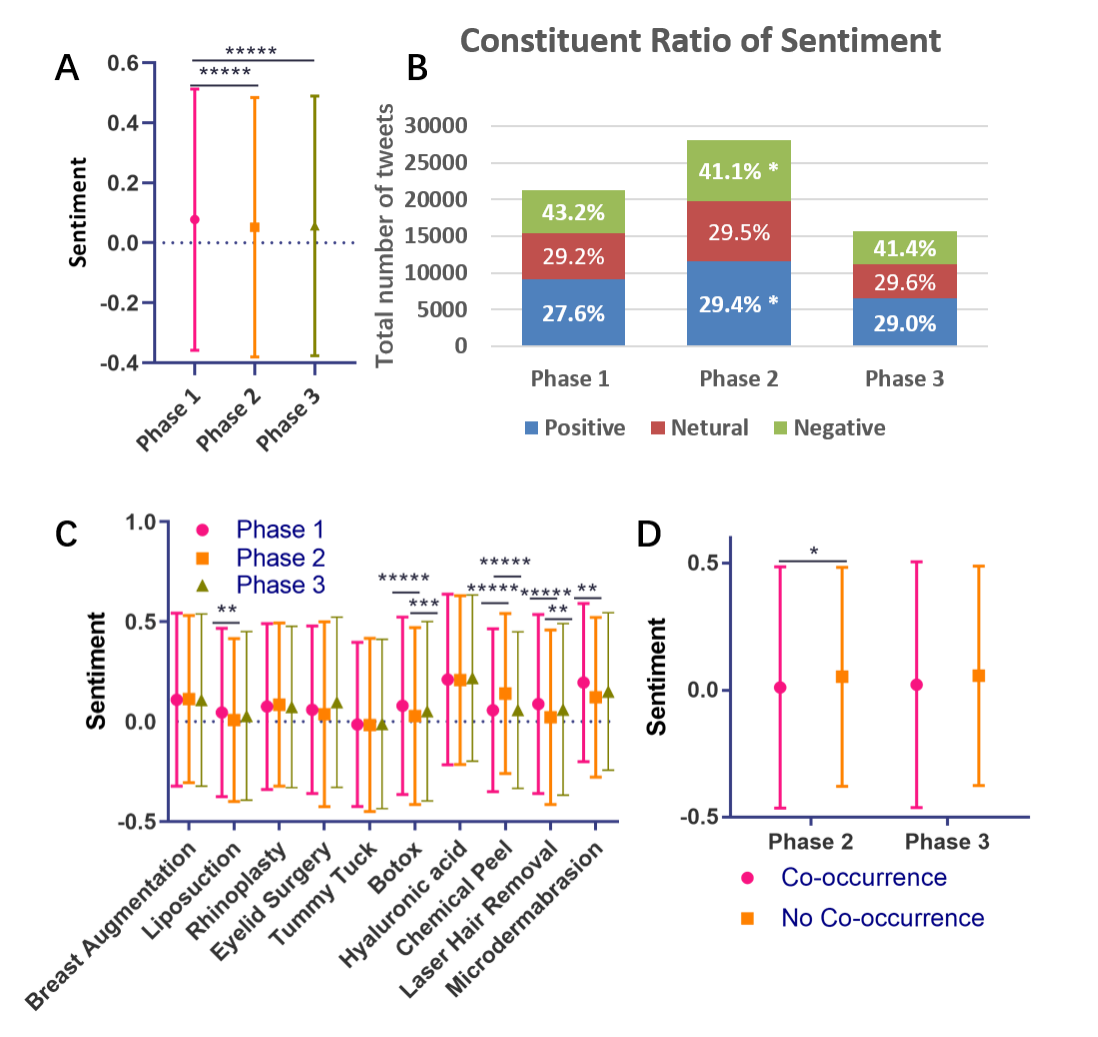
(A) Overall tweet sentiments in the three different phases. A negative number indicates negative sentiment, and a positive number indicates positive sentiment. (B) Total numbers and constituent ratios (labels in cells) of sentiments in different phases. (C) Sentiments for specific procedures. (D) Sentiments for tweets in which mentions of the procedures do or do not co-occur with mentions of COVID-19 in phases 2 and 3. *P<.05, **P<.01, ***P<.001.

In addition to tweeting frequency, sentiment can serve as an indicator of public interest toward plastic procedures. As shown in Figure 4A, in general, the sentiments of tweets became more negative in phase 2 and showed no sign of rebounding in phase 3. A constituent ratio analysis found higher negative sentiment proportions and lower positive proportions in phase 2 and phase 3 than in phase 1 (Figure 4B), which is in line with the overall results. The sentiment analysis for specific procedures found that sentiments about *Botox* and *laser hair removal* decreased first and then rebounded, while sentiments about *chemical peel* showed the opposite trend (Figure 4C).

We further analyzed tweets in which *covid* or *coronavirus* co-occurred with mentions of plastic procedures, and we found that in phase 2, the sentiment of these tweets was more negative than that of tweets that did not mention COVID-19; meanwhile, this difference was not statistically significant in phase 3 (Figure 4D).

### Word Frequency

**Figure 5:**
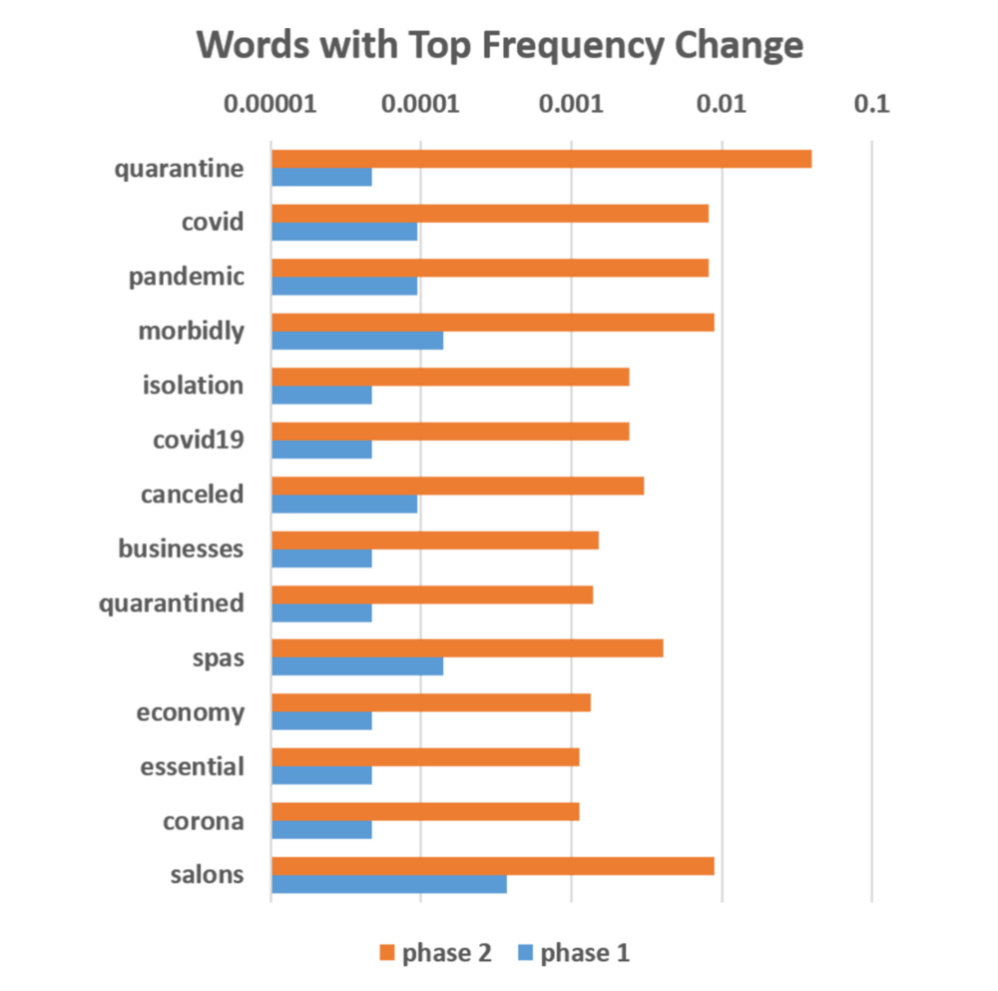
Frequencies of words with the greatest frequency changes.

Word frequency can reflect the trending of topics, and changes in frequently used words can reveal a shift in public interest to some extent. The words with the highest frequency changes in phase 2 versus phase 1 are shown in Figure 5. It should be noted that Figure 5 was slightly manually revised to exclude some words contaminated by Twitter bots.

The increased frequency of the words *quarantine*, *covid*, *pandemic*, *isolation*, *covid19*, *quarantined*, and *corona* indicated that COVID-19 became a major concern among people who were interested in plastic procedures. Words including *canceled*, *spas*, and *salons* usually co-occurred in phase 2. In fact, *canceled* also usually co-occurred with mentions of plastic procedures; this is not indicated in Figure 5 because this frequency change was small. The co-occurrence of these words indicates that many appointments related to plastic procedures were canceled due to the COVID-19 pandemic. Table 1 provides some typical related tweets (tweets 1 and 2). The term *morbidly* ranked high because of the surge of the hashtag #morbidlyobese. The frequency of the terms *essential* and *business* increased because many cosmetic services were considered nonessential and were cancelled, as shown in Table 1 (tweets 3 and 4). The increased frequency of the word *economy* demonstrates the public concern about the economy.

**Table 1:** Typical tweets related to plastic procedures. Only the tweets and their publication dates are provided to protect the privacy of their writers. The tweet contents were minimally revised to remove some foul language.

No	Date (2020)	Tweet
1	March 20	“My mom said she didn’t take this pandemic serious till they canceled her laser hair removal, now she’s stocking up”
2	March 20	“I was supposed to get a chemical peel today but of course that was canceled. I just wanted to have perfect skin while quarantined but I guess that’s too much to ask for.”
3	April 17	“I wanted to get my lips and Botox redone but apparently med spas aren't essential”
4	April 20	“Not a day goes by in which I don’t feel pain that I wasn’t able to get a chemical peel before non-essential businesses closed”
5	May 11	“This is the perfect time for a chemical peel. I’ll at least step out for one once salons get back running”
6	May 22	“This would have been the perfect time to heal from some Botox n filler”
7	April 27	“I HATE SHAVING! The worst part of quarantine is not being able to go to my laser hair removal procedures. I was almost done with my 6 months, now I have to start all over. Shaving hurts like hell too, I'm pissed.”
8	2020/4/23	“Influencers Are Still Getting Lip Fillers and Botox During Lockdown - Even during COVID-19's stay-at-home measures, spas and clinics are offering Botox and fillers at a reduced rate to influencers — and some are taking the riskier route of doing i“
9	2020/5/2	“the med spas are having covid botox sales. Brb”

The word frequencies between phase 2 and phase 3 were also compared. Some frequently appearing words are related to celebrities, such as *Kamala Harris* and *Kellyanne Conway*. Some other words are related to the Black Lives Matter movement, such as *Floyd* and *racist*. These results are not closely related to the aim of this study; therefore, they are not shown. Further details are provided in Multimedia Appendix 2. It should be noted that words related to plastic procedures, although they showed no significant frequency changes, remained the most common; this can be observed in the word cloud figure for phase 3 in Multimedia Appendix 3.

## Discussion

### Principal Findings

In this study, we performed a survey of 73,963 publicly accessible tweets about the most common plastic procedures from January 1 to July 22, 2020. By integrating tweeting frequency, sentiment, and word frequency analyses, we aimed to depict changes in public interest toward these plastic procedures and help stakeholders in the field of plastic surgery to sustain their operations during the difficult time of the COVID-19 pandemic. To the best of our knowledge, this study is the first to address this issue.

In this study, the keywords to be searched on Twitter were determined by referring to the annual Plastic Surgery Statistics published by the American Society of Plastic Surgeons [16]. The top five cosmetic surgical procedures and top five minimally invasive procedures certainly do not represent all plastic procedures. However, an exhaustive survey of all plastic procedures is unfeasible. The criteria used to determine which procedures to include or exclude may be controversial. Furthermore, Twitter users may use nonstandard expressions. However, these top procedures should cover most of the daily practices of many stakeholders. For the procedures that were not included, readers can refer to our methods and determine their own findings. Additionally, readers are welcome to contact us, and we will try our best to assist them.

The definition of different groups of tweets was quite difficult, as the pandemic did not occur at one static time point and evolved rapidly. There was no gold standard or even related research we could refer to. By scrutinizing trends in tweeting frequency and Google Trends indices, we found that the data fluctuated drastically around the time points of the WHO’s declaration of the COVID-19 pandemic and the death of George Floyd. Naturally, we divided the collected data into three phases based on these two landmark events. The results of the tweeting frequency, sentiment, and word frequency analyses show good discrimination among these three phases. Therefore, the definition of the three phases should be suitable. There are complicated epidemiological, economic, and political reasons why these data showed distinct features in the three phases. However, these reasons are not of concern to this study; therefore, we left this question unaddressed.

In phase 2, the tweeting frequency, tweet sentiments, and word frequency all changed significantly. The tweeting frequency increased by up to 24.0%, which was somewhat surprising at first sight. Further analysis indicated that the increase was mainly contributed by the terms *Botox* and *chemical peel* (Figure 3B). Scrutiny of the related tweets revealed that many people saw the quarantine as a perfect time to receive Botox and chemical peels, as shown in Table 1 (tweets 5 and 6). This may be due to the increased free time and decreased exposure to others due to quarantine. The tweeting frequencies of most other procedures also increased (Figure 3B). Therefore, public interest in plastic procedures generally increased in phase 2, which may represent a benefit for plastic surgery stakeholders during the pandemic. However, it should be noted that the increase in public interest does not necessarily indicate that more plastic procedures were performed during this phase.

The subsequent sentiment analysis indicated that the COVID-19 pandemic resulted in negative sentiment regarding public interest in plastic procedures. However, the negative sentiment was not always detrimental for plastic surgery stakeholders. As shown in tweet 7 in Table 1, in one of the most negative tweets in phase 2, the writer expressed a strong desire for laser hair removal. The subsequent word frequency analysis basically reflected the confusion created by the COVID-19 pandemic among the public.

Upon moving to phase 3, the changes appeared to be less substantial. The overall tweeting frequency decreased but was still higher than that in phase 1. Not surprisingly, this decrease was mainly contributed by decreases in the frequency of the terms *Botox* and *chemical peel* (Figure 3B). This result may not indicate that the public was losing interest in these procedures but may be due to the decreased demand due to the restarting of the economy. In fact, people may have undergone more Botox and chemical peel procedures in phase 3 than in phase 2, which may be supported by the rebounding search indices in Google Trends (Figure 2). The discrimination between the tweeting frequency and Google Trends indices data may lie in their different natures: people express their thoughts and sentiments on Twitter, while Google is more of a tool to which people resort when they are about to take action. Our results suggest that Botox and chemical peel procedures will be the first to revive once lockdown is lifted. Because the prediction of the second wave of COVID-19 is not simply due to paranoia [28,29], stakeholders in the plastic surgery field should be prepared in case additional lockdowns are deployed.

The number of tweets that mentioned COVID-19 decreased in phase 3 (Figure 3D), and no overall sentiment difference was found (Figure 4 A, B, and D). There is also no major word frequency difference regarding plastic procedures in phase 3 versus phase 2. It appears that the public had become accustomed to coexisting with COVID-19, and their interest in plastic procedures did not decrease.

At the micro level, the tweeting frequencies of *breast augmentation*, *tummy tuck*, and *laser hair removal* increased in phase 3, and the frequencies of the first two terms also increased in phase 2. In addition to the overall tweeting frequency, the constituent ratio should also be considered. Because the damaged economy may narrow consumers’ choices, plastic procedures may be required to compete with each other for consumers’ favor. The constituent ratios of *liposuction* and *tummy tuck* consecutively increased in both phases 2 and 3, while the constituent ratios of *breast augmentation*, *laser hair removal*, and *eyelid surgery* only increased in phase 3. Taken together, the breast augmentation, liposuction, and tummy tuck procedures surpassed other procedures, as they showed a better absolute or relative increase. This result is not surprising, as breast augmentation and liposuction are the most popular cosmetic surgical procedures according to the latest annual Plastic Surgery Statistics published by the American Society of Plastic Surgeons [16]. Tummy tuck may not have ranked as highly as the other two procedures; however, this procedure may benefit from the effects of the current pandemic on people’s lifestyles. People may be required to stay at home due to quarantine or be unable to afford to exercise at the gym due to economic concerns. Therefore, they have a higher likelihood of becoming overweight and may seek a tummy tuck or liposuction.

In summary, plastic surgery stakeholders should consider refocusing on breast augmentation, liposuction, and tummy tuck procedures at the current stage of the pandemic. If a second wave of COVID-19 occurs, stakeholders should prepare for a temporary surge of Botox and chemical peels. However, this does not mean that other procedures are unimportant, and they are still included in the majority of all plastic procedures.

When scrutinizing our data, we found that many stakeholders tried various strategies to survive the difficult period of the COVID-19 pandemic. As shown in Table 1 (tweets 8 and 9), they made use of the influence of key opinion leaders and provided more flexible prices. These efforts are praiseworthy and should be adopted by others.

### Limitations

The major limitation of this study is that the “real world” is much more complicated than reflected by tweet data, even though the study is based on as many as 73,963 tweets and the research methods are well established. All surveys based on web-based social media platforms face this problem. Furthermore, the COVID-19 pandemic is unprecedented in many aspects. Because this is the first and only study of its kind to date, we did not have many other studies to refer to. Therefore, the results are open to wiser explanation by readers. Due to the limitation of Twitter privacy settings, we could not perform more precise analyses based on age, gender, or location. Additionally, the impact of the COVID-19 pandemic could vary drastically in different districts and at different time points. Readers should apply the results of this study at their own risk.

### Conclusions

The public has maintained their interest in plastic procedures during the COVID-19 pandemic. Stakeholders in the field of plastic surgery should consider refocusing on breast augmentation, liposuction, and tummy tuck procedures at the current stage of the pandemic. In case of a second wave of COVID-19, stakeholders should prepare for a temporary surge in requests for Botox and chemical peels.

## Appendix

Multimedia Appendix 1 Original tweet data. Only tweets and publication dates are provided to protect the tweet writers' privacy.

Multimedia Appendix 2 Word frequencies in all three phases.

Multimedia Appendix 3 Word cloud of tweets in phase 3. The size of each word is in proportion to its frequency.

## Abbreviations

NLTK: Natural Language Toolkit
VADER: Valence Aware Dictionary and Sentiment Reasoner
WHO: World Health Organization
