# Supplementary figures and images for: Public Interest in Cosmetic Surgical and Minimally Invasive Plastic Procedures During the COVID-19 Pandemic: Infodemiology Study of Twitter Data
Source: J Med Internet Res. 2021 Mar 16;23(3):e23970. doi: 10.2196/23970 (PMC7968479; doi:10.2196/23970)

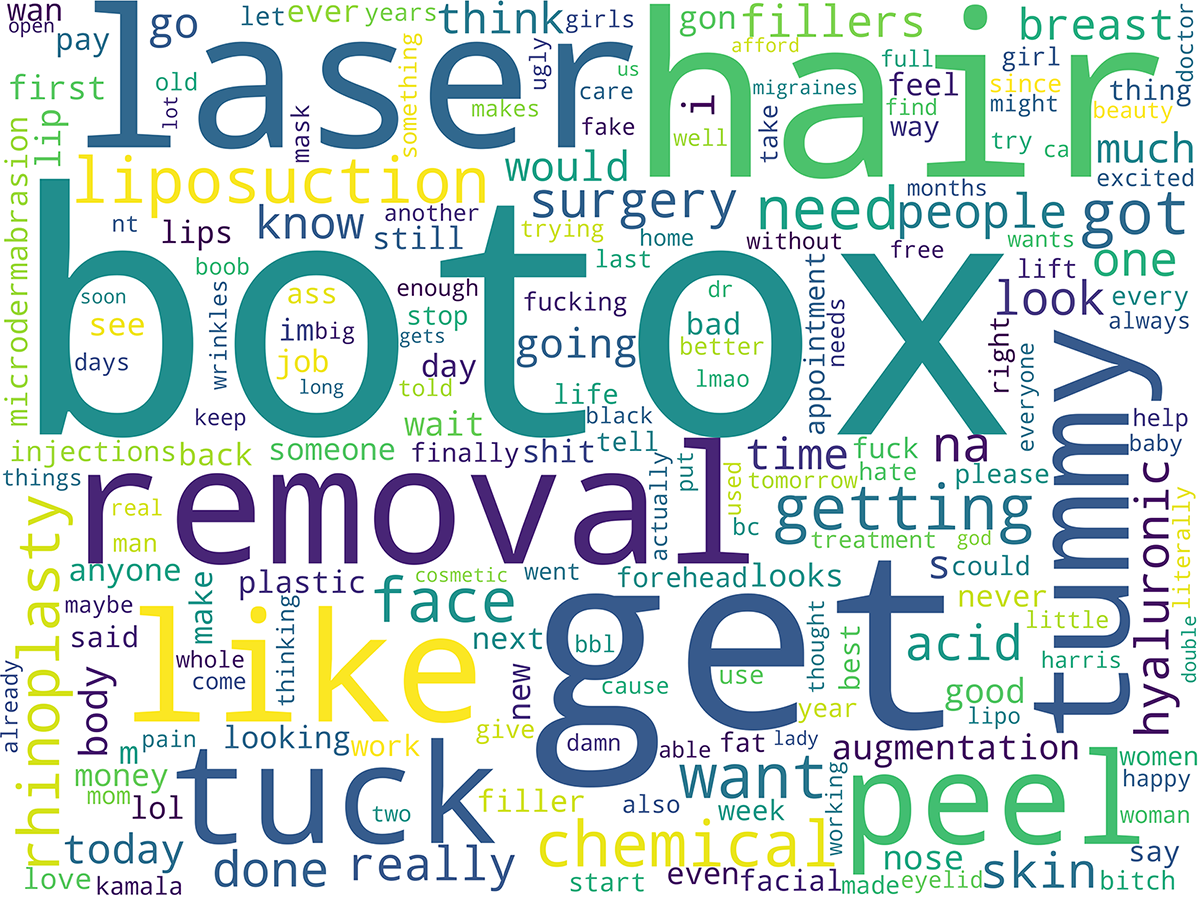

Supplement: Multimedia Appendix 3 [file jmir_v23i3e23970_app3.png]
